# Supplementary material for: Microbial biofilms for electricity generation from water evaporation and power to wearables
Source: Nat Commun. 2022 Jul 28;13:4369. doi: 10.1038/s41467-022-32105-6 (PMC9334603; doi:10.1038/s41467-022-32105-6)
Supplement: Supplementary file 1 — Supplementary Information [file 41467_2022_32105_MOESM1_ESM.pdf]

Supplementary Information for

Microbial Biofilms for Electricity Generation from Water Evaporation and Power to Wearables

Xiaomeng Liu[1], Toshiyuki Ueki,[2] Hongyan Gao[1], Trevor L. Woodard,[2] Kelly P. Nevin,[2] Tianda Fu,[1] Shuai Fu,[1] Lu Sun,[1] Derek R. Lovley[2,3]*, Jun Yao[1,3,4]*

1. Department of Electrical Computer and Engineering, University of Massachusetts, Amherst, MA, USA.
2. Department of Microbiology, University of Massachusetts, Amherst, MA, USA.
3. Institute for Applied Life Sciences (IALS), University of Massachusetts, Amherst, MA, USA.
4. Department of Biomedical Engineering, University of Massachusetts, Amherst, MA, USA.

* Corresponding authors. Emails: juny@umass.edu (J.Y.); dlovley@umass.edu (D.R.L.)

This file includes:

Supplementary Figures S1 – S22

Supplementary References

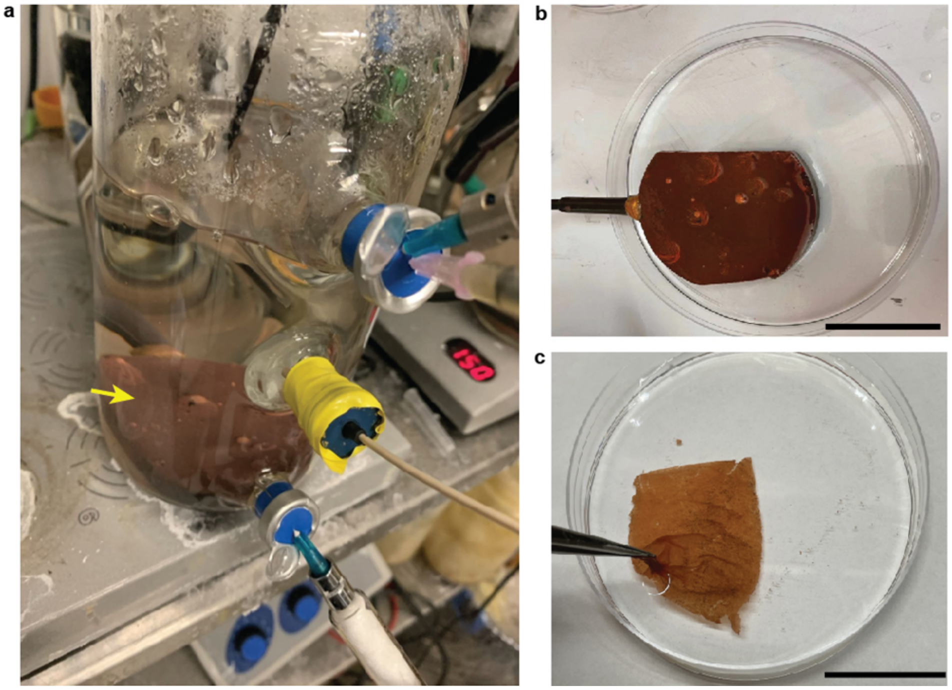

Supplementary Figure S1. **Biofilm growth and harvesting**. **a**, Setup for *G. sulfurreducens* biofilm growth. The arrow indicates the growth electrode. Details about the growth process can be found in *Methods*.[1] **b**, As-grown *G. sulfurreducens* biofilm on a polished graphite electrode. Scale bar, 5 cm. **c**, A harvested biofilm floating on water. The biofilm was harvested by scraping it off the electrode with a blade, rinsed in water, and stored in a 4 °C refrigerator. Scale bar, 5 cm.

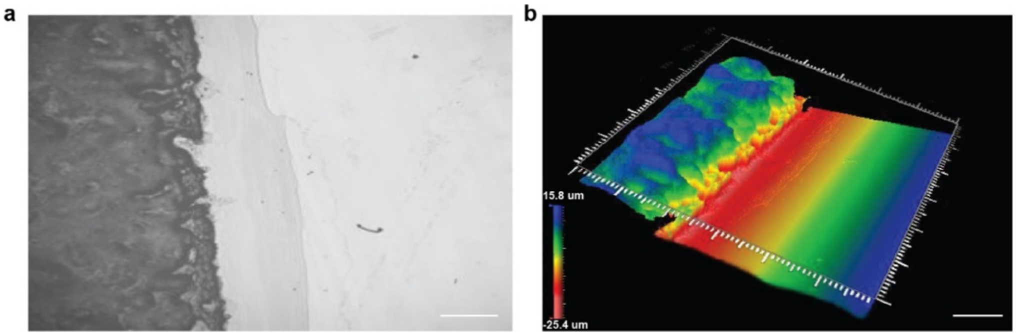

Supplementary Figure S2. **Measurement of biofilm thickness**. **a**, Optical image of a *G. sulfurreducens* biofilm (dark region) prepared on a silicon substrate. Scale bar, 1 mm. **b**, 3D topological image of the biofilm by using a non-contact 3D profiler (NewView™ 9000; Zygo). Scale bar, 1 mm. The measured thickness was 38 ± 3 µm. The biofilm was kept wet during the imaging to reflect the thickness under device working condition.

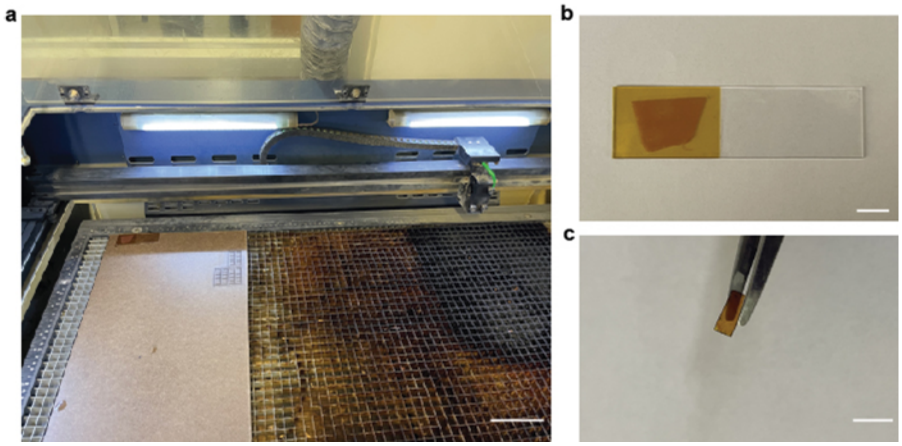

Supplementary Figure S3. **Biofilm patterning by laser writing**. **a**, The laser writing system (LaserPro Spirit GLS; GCC). Scale bar, 10 cm. **b**, An as-prepared *G. sulfurreducens* biofilm before patterning. The biofilm was placed on polyimide (PI) substrate further supported by a glass slide (for easy transfer). Scale bar, 1 cm. **c**, A patterned biofilm by the laser writer (15% power, Speed 20%, 400 points per inch resolution). Scale bar, 1 cm.

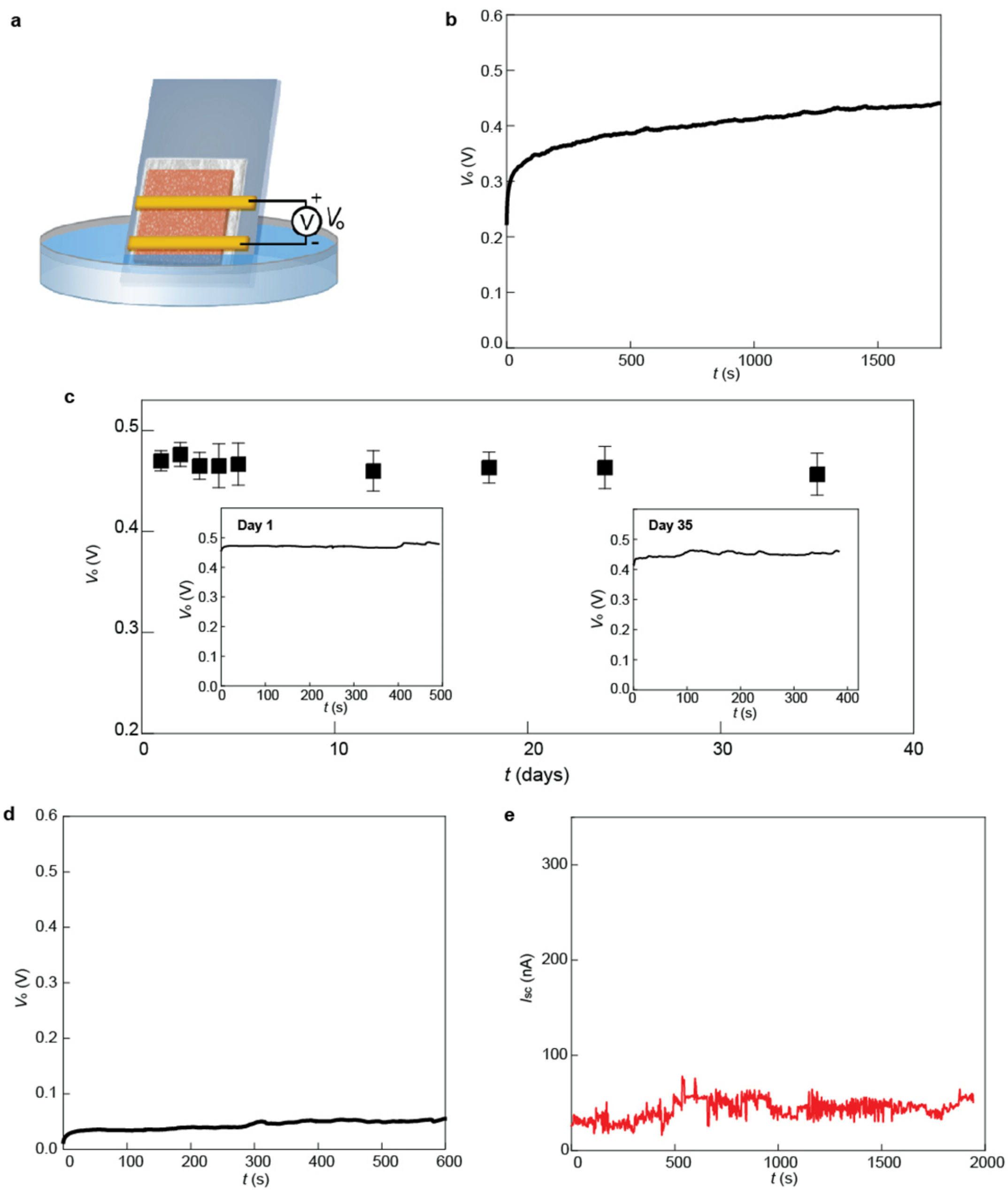

Supplementary Figure S4. **Electric output from *G. sulfurreducens* biofilm device and control**. **a**, Schematic of the biofilm device used in Fig. 1d in the main text. The biofilm was placed on a tissue paper supported by a glass slide (25 × 75 mm^2). A pair of electrodes (2 × 15 mm^2), made from Au-coated polyimide films, was covered on top of the biofilm. The tissue paper facilitated the water transport but did not contribute substantially to energy output as shown in (d) and (e). Devices without tissue paper produced the same effect (Supplementary Fig. S7). **b**, The measured open-circuit voltage (V_o) from a biofilm device. The size of the biofilm device was 2 × 10 mm^2. **c**, A continuous 35-day recording of the V_o from the device. The insets show representative recording curves on day-1 and day-35, respectively. The error bars are standard deviations. **d**, Voltage output (V_o ~0.05 V) from the tissue paper after removing biofilm. **e**, Short-circuit current output (I_{sc} ~30 nA) from the tissue paper after removing the biofilm. The measurements were

performed in the ambient environment with a relative humidity (RH) of ~50%. All the error bars are standard deviations.

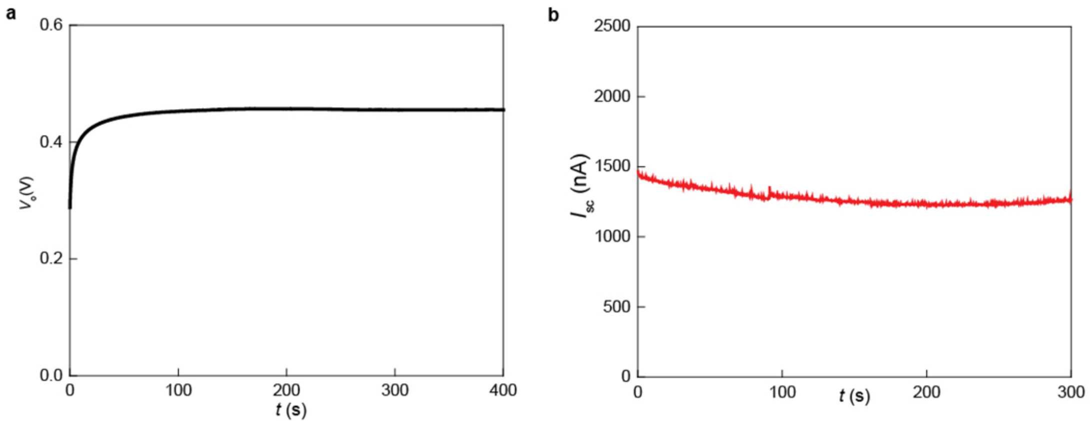

Supplementary Figure S5. **Biofilm device using carbon electrodes**. **a**, Open-circuit voltage (V_o) measured from a *G. sulfurreducens* biofilm device having the same structure as shown in Supplementary Fig. S4, with the pair of Au electrodes replaced by a pair of carbon electrodes. The carbon electrodes were defined by printing conductive carbon ink on polyimide stripes (Dimatix Inkjet DMP 2831; Fujifilm). **b**, Measured short circuit current (I_{sc}) of the device. The measurements were performed in the ambient environment with a RH ~50%. Both results were close to values obtained with Au electrodes.

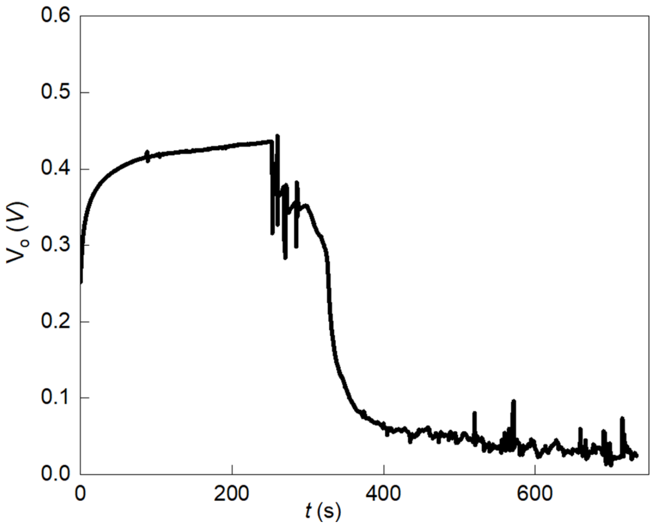

Supplementary Figure S6. Voltage output (V_o) from a biofilm by pulling it out from the water. The device had the same structure as shown in Supplementary Fig. S4.

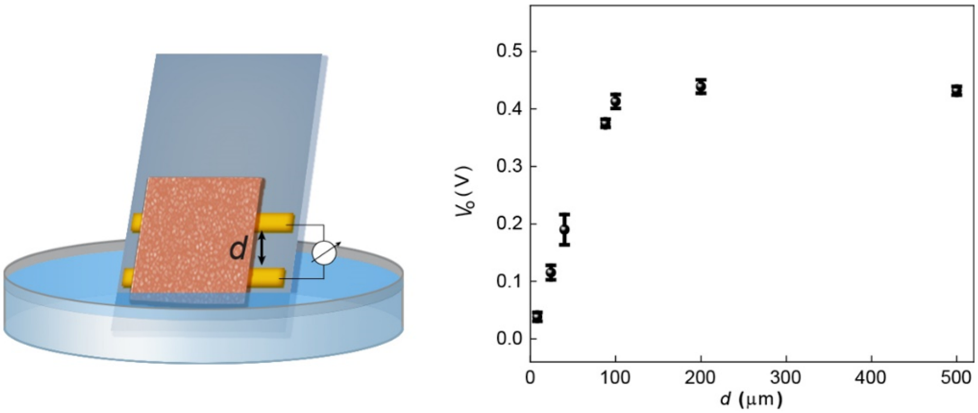

Supplementary Figure S7. **Dependence of voltage output on electrode spacing.** The open-circuit voltage outputs (V_o) were measured in biofilm devices defined with different electrode spacings (d) of 8, 24, 40, 88, 100, 200, 500 μm by immersing one terminal in water (left schematic). V_o linearly increased with the increase in d and saturated at d~100 μm (right panel). The short fluidic distance of ~100 μm was consistent with the fast evaporation rate in the biofilm (Supplementary Fig. S13). The electrodes (Ti/Au 5/50 nm, 4 μm × 9 mm size) were lithographically defined on a Si substrate covered with 600 nm SiO_2 (Nova Electronic Materials). The biofilm (5×2 mm^2) was transferred onto the pair of electrodes. The measurements were performed in ambient environment with RH~30%. The error bars are standard deviations.

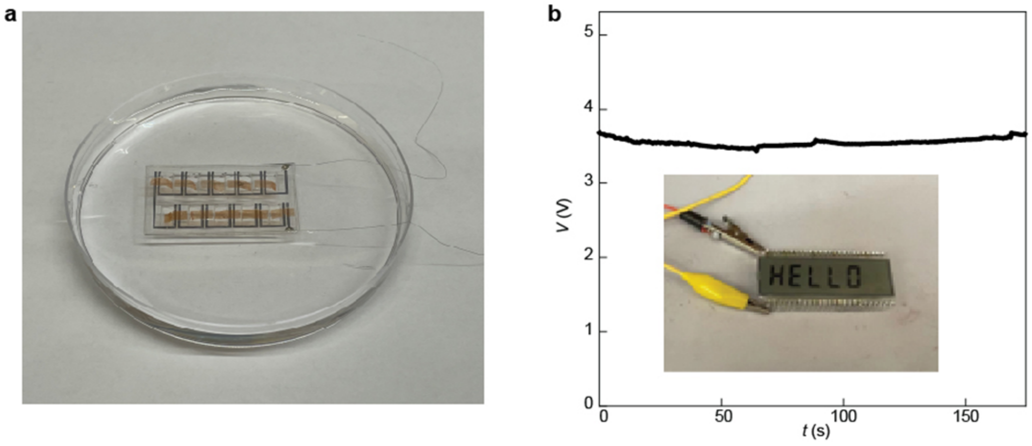

Supplementary Figure S8. **Interconnected biofilm device array**. **a**, 10 biofilm devices were connected in series on a polyethylene terephthalate (PET, ~80 µm thick) substrate, with the PET substrate further placed on a polydimethylsiloxane (PDMS, ~0.5 mm thick) substrate floating on water (device structural schematic can be found in Fig. 1b-ii in the main text). The biofilms and openings on PET substrate were patterned with a laser writer (LaserPro Spirit GLS; GCC). The spacing of each pair of electrodes was 1 mm and the size of each biofilm was ~3×7 mm^2. **b**, Voltage output (*V*) measured from the device array, which was used to power a LCD (VI-602-DP-FC-S; Varitronix) as shown in inset.

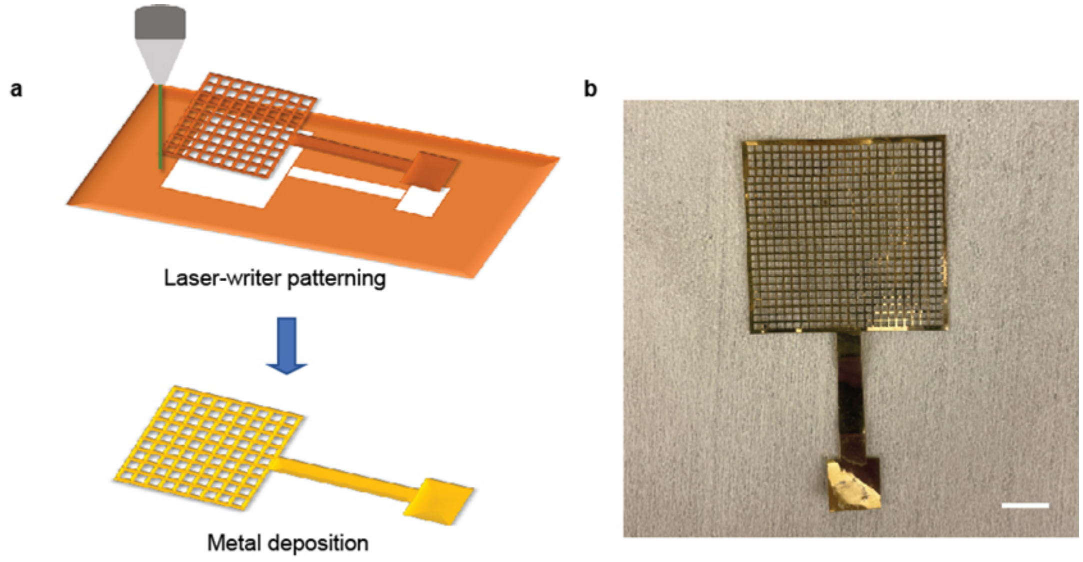

Supplementary Figure S9. **Fabricating mesh electrode**. **a**, Schematics of fabricating the mesh electrode, involving (top) patterning a PI substrate using a laser writer (LaserPro Spirit GLS; GCC) and (bottom) coating the defined PI mesh with a metal layer (Cr/Au = 5/50 nm) using standard metal deposition. **b**, An as-fabricated mesh electrode. Scale bar, 5 mm.

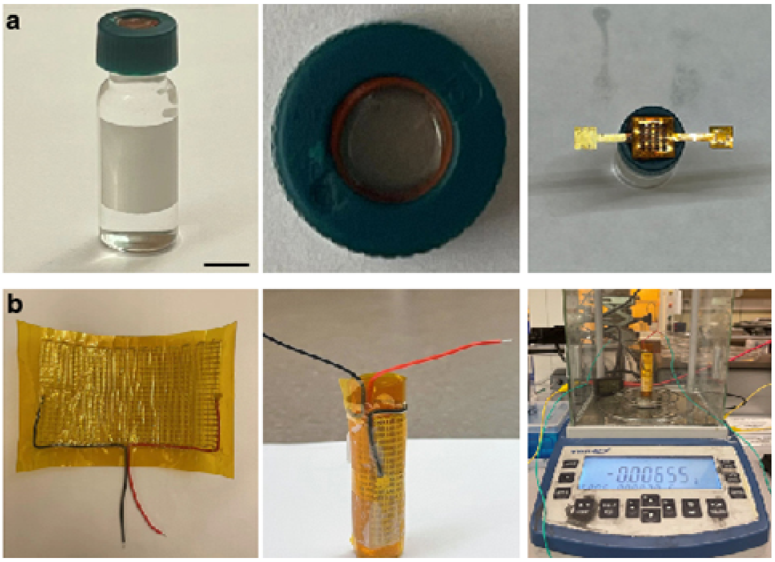

Supplementary Figure S10. **Measurement of evaporation rate across the biofilm**. (**a**) A biofilm device was used to seal an autosampler vial fully filled with water (left) by covering the device on the opening of the lid (middle). Scale bar, 5 mm. (**b**) The vial was coated with a flexible heating pad (left) to control the water temperature. The power in the heating pad was calibrated by measuring the water temperature with a thermal meter. The vial was placed on a digital weight gauge (VWR-225AC, 0.01 mg resolution) so that the water loss by evaporation across the biofilm could be monitored (right). Corresponding electric outputs from the biofilm device were measured under the same conditions.

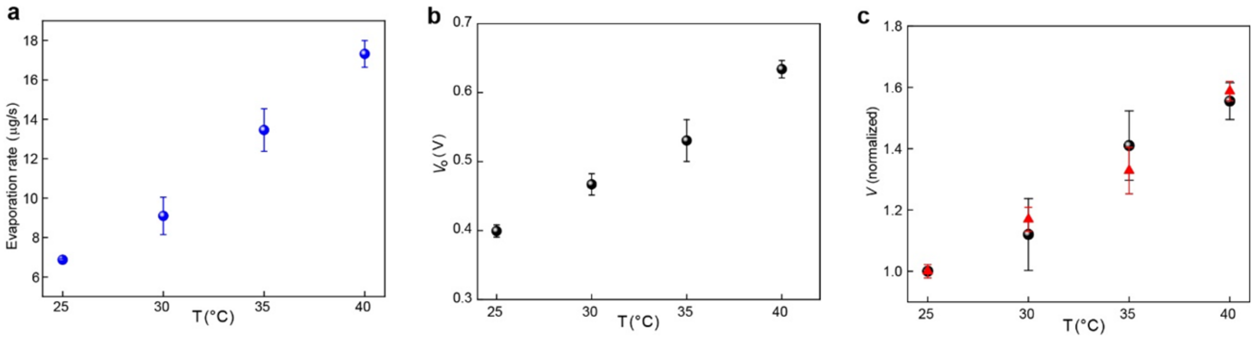

Supplementary Figure S11. **Evaporation rate and voltage output**. (**a**) Evaporation rates across the biofilm device (5×5 mm^2 size, 0.4 porosity, 100 μm pore size) at different water temperatures measured by the experimental setup in Supplementary Fig. S10. (**b**) Corresponding measured open-circuit voltage (V_o) from the device at different temperatures. (**c**) Comparison between the trend of measured voltage (black) and the predicted trend of streaming potential (red). All the error bars are standard deviations.

To obtain the predicted trend, the expression of streaming potential in a standard microfluidic channel was used:[2]

$$V_s = \frac{\varepsilon_0 \varepsilon_r \Delta P \zeta}{\sigma \eta} \text{----------------------------(1)},$$

where $\varepsilon_0\varepsilon_r$, σ, η, ζ, and ΔP are the permittivity, conductivity, viscosity of the water solution, *zeta* potential of the channel surface, and pressure difference across the channel. According to general fluid dynamics,[3] ΔP is proportional to the evaporation rate measured in (**a**). The temperature-dependent values of $\varepsilon_0\varepsilon_r$, σ, η were obtained from refs. 4-6, respectively. These values were normalized as shown in table below. Temperature effect on the material *zeta* potential[2] was not considered here.

T (°C)	25	30	35	40	Ref.
ΔP	1	1.32	1.96	2.52	Based on (**a**)
$\varepsilon_0\varepsilon_r$	1	0.97	0.95	0.93	4
σ	1	1.28	1.63	2.08	5
η	1	0.90	0.81	0.73	6

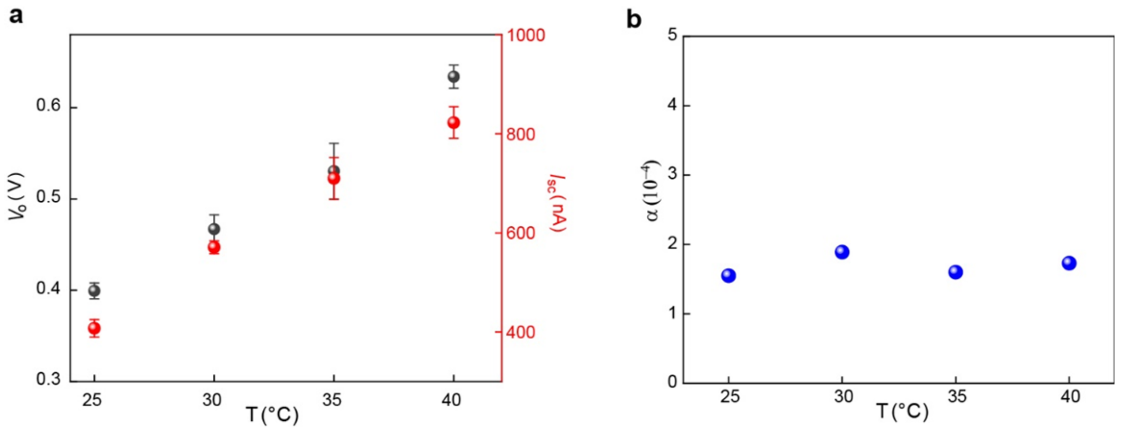

Supplementary Figure S12. **Energy conversion efficiency**. (**a**) Measured open-circuit voltage (V_o) and short-circuit current (I_{sc}) from the biofilm device (5×5 mm^2 size, 0.4 porosity, 100 μm pore size) used in the experimental setup shown in Supplementary Fig. S10 at different temperatures. (**b**) Calculated energy conversion efficiency α based on measured parameters. All the error bars are standard deviations.

Specifically, the conversion efficiency is defined as the ratio between the output electric energy and the input kinetic energy. The input kinetic power $P_{kinetic}$ can be calculated as $P_{kinetic} = Q \cdot \Delta p$, where Q is the volume flow rate and Δp the pressure difference across the biofilm.[7] The optimal output electric energy can be approximated as $P_{electric} = V_o \cdot I_s / 4$, where V_o and I_s are the measured open-circuit voltage and short-circuit current (as shown in **a**), respectively. Therefore, the conversion efficiency α can be obtained as:

$$\alpha = \frac{P_{electric}}{P_{kinetic}} = \frac{V_o \cdot I_s}{4 \cdot Q \cdot \Delta p} \text{ ----------------------(2)}$$

The volume flow rate can be obtained as $Q = \Delta m / \rho$, where Δm, ρ are the measured weight loss rate (Supplementary Fig. S11a) and saturated vapor density,[8] respectively.

Δp is the difference of vapor pressure across the biofilm device. The vapor pressure inside the vial can be treated as saturated value, whereas the vapor pressure at the outer side of biofilm can be directly measured. For example, at the water temperature of 25 °C, the relative humidity (RH) and temperature outside the vial at the vicinity of the biofilm device were measured (SEK-SHT40-AD1B-Sensors; Sensirion) to be ~86% and 22 °C, respectively, whereas the RH and temperature inside the vial were treated as 100% and 25 ºC, respectively. The vapor pressure can be determined with given temperature and RH.[9] Consequently, $\Delta p_{25\,°C} = p_{100\%RH,25\,°C} - p_{86\%RH,22°C} = 8.8 \times 10^2$ Pa. According to general fluid theorem[3] that the volume flow rate is proportional to Δp, Δp_T at other temperature was obtained by $\Delta p_T = (\Delta m_T / \Delta m_{25\,°C} \cdot \Delta p_{25\,°C})$, where Δm is the measured evaporation rate (Supplementary Fig. S11a).

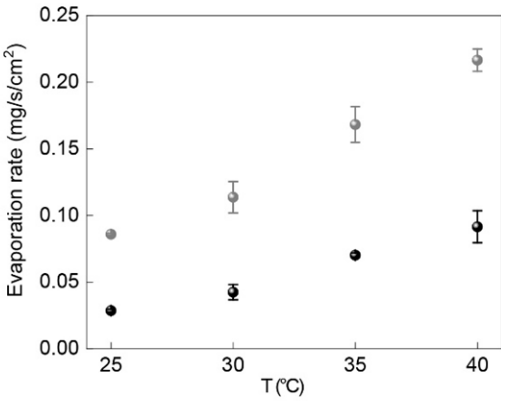

Supplementary Figure S13. **Comparison of evaporation rates**. Water evaporation from the vial (see setup in Supplementary Fig. S10) through the open lid (black dots) were slower rate than through the lid covered with a biofilm device (gray dots). Note that since the biofilm device had a porosity of 0.4, a factor of 2.5 was multiplied for calculating its areal rate. All the error bars are standard deviations.

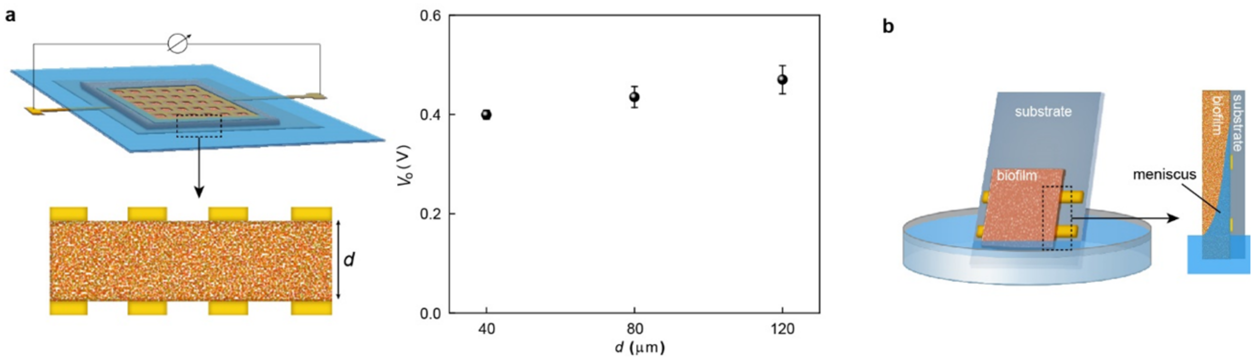

Supplementary Figure 14. **Dependence of voltage output on biofilm thickness**. **a**, The open-circuit voltage outputs (V_o) measured from devices made of monolayer, double-layer, and triple-layer biofilm-sheets (left schematic). The biofilm established the (saturating) voltage across the vertical thickness with a distance (*e.g.*, ≤ 40 μm) even shorter than that along the in-plane direction (~100 μm, Supplementary Fig. S7), probably because of the meniscus effect[10] at the biofilm-substrate interface in the planar device (**b**) extends the fluidic length. The error bars are standard deviations.

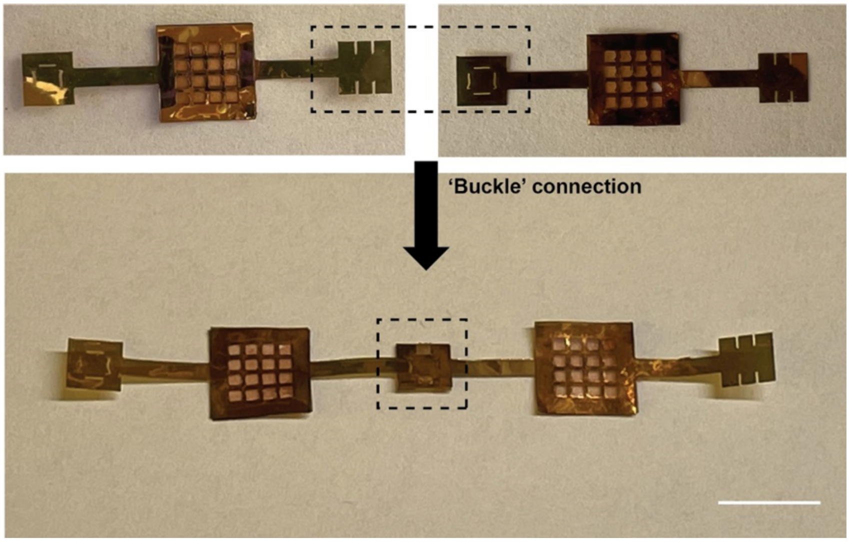

Supplementary Figure S15. **The design of a 'buckle' connection**. The contacts of top and bottom *mesh* electrodes were patterned with socket and plug features by laser writing as shown in the top panel, which enabled an easy buckle connection between the devices (bottom). Note that the design also allowed for a 90-degree rotation in the connection. Scale bar, 5 mm.

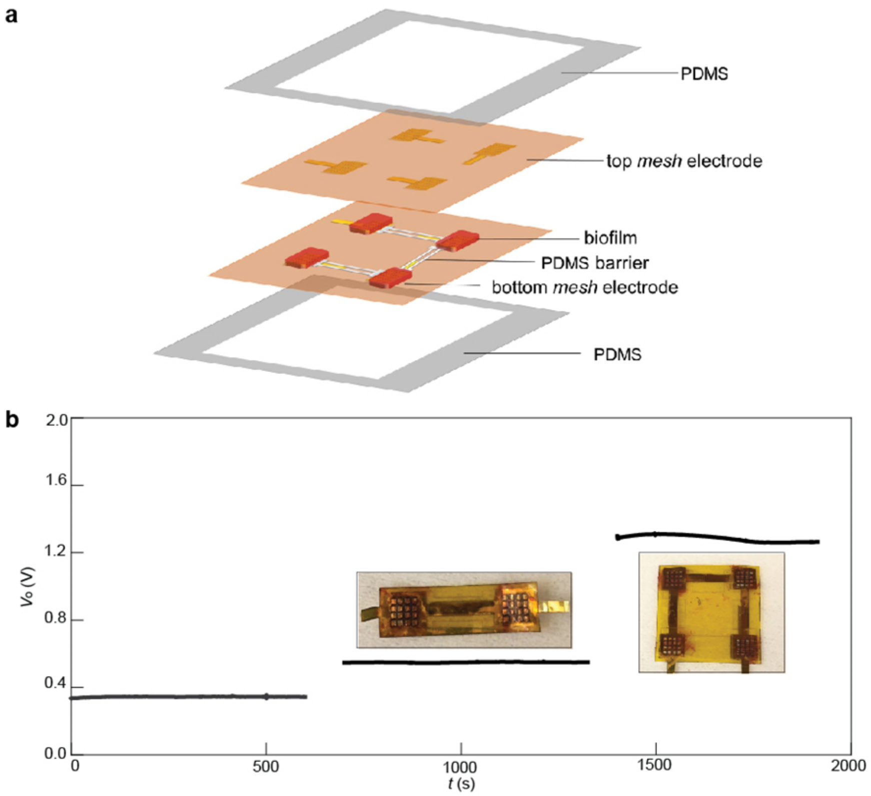

Supplementary Figure S16. **On-substrate integration of biofilm devices**. **a**, Schematic of series integration of biofilm devices. The top and bottom *mesh* electrodes were defined on polyimide films using a laser writer, followed by selected-area metal deposition (Cr/Au = 5/50 nm) using a shadow mask. The contacts of top and bottom mesh electrodes were arranged in a way that they would form series connection when put into contact. Patterned biofilms were then transferred onto the bottom mesh electrodes. A molded PDMS barrier layer (~20 µm thick) was sandwiched between the top and bottom electrodes to prevent short circuit at the biofilm edge. The device was then sealed by two PDMS layers (~20 ×µm thick). **b**, Open-circuit voltage (V_o) measured from fabricated devices containing 1, 2, and 4 biofilms, showing linear increase in output.

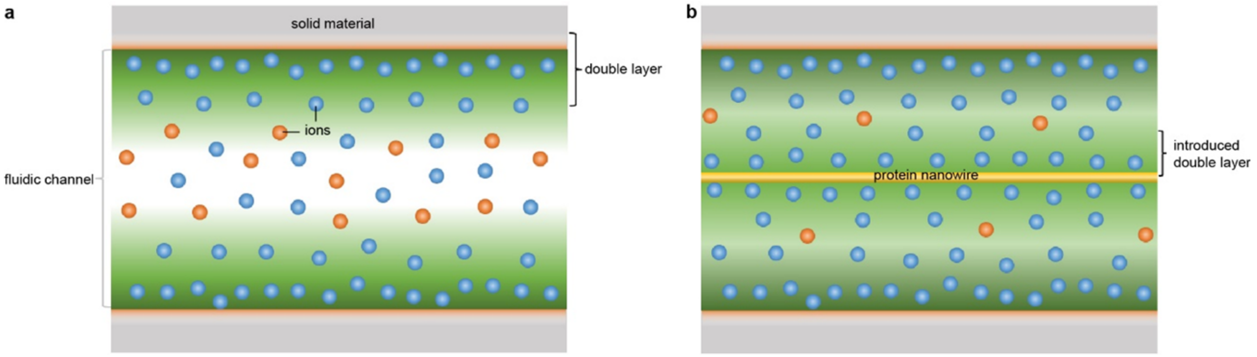

Supplementary Figure S17. **Double-layer overlap**. **a**, Schematic of a fluidic channel having minimal overlap in double layers. **b**, A protein nanowire introduces an additional water-solid interface and double layer, which increases the overlap in double layers.

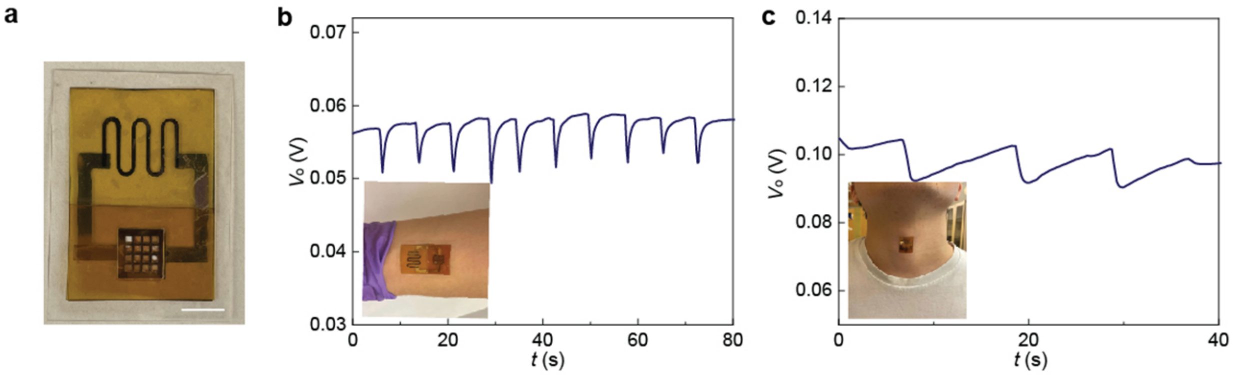

Supplementary Figure S18. **Integrated sensor and biofilm for wearable detection**. **a**, An integrated wearable patch with a crack strain sensor[11] directly connected to the biofilm device. Details of fabrication can be found in *Methods*. Scale bar, 5 mm. **b**, Measured bodily mechanical signal when bending the wrist using the wearable patch. **c**, Measured bodily mechanical signal of swallowing using the same patch. The signals were acquired by connecting the two terminals of the crack sensor to a source meter (Keithley 2401; Keithley Instruments).

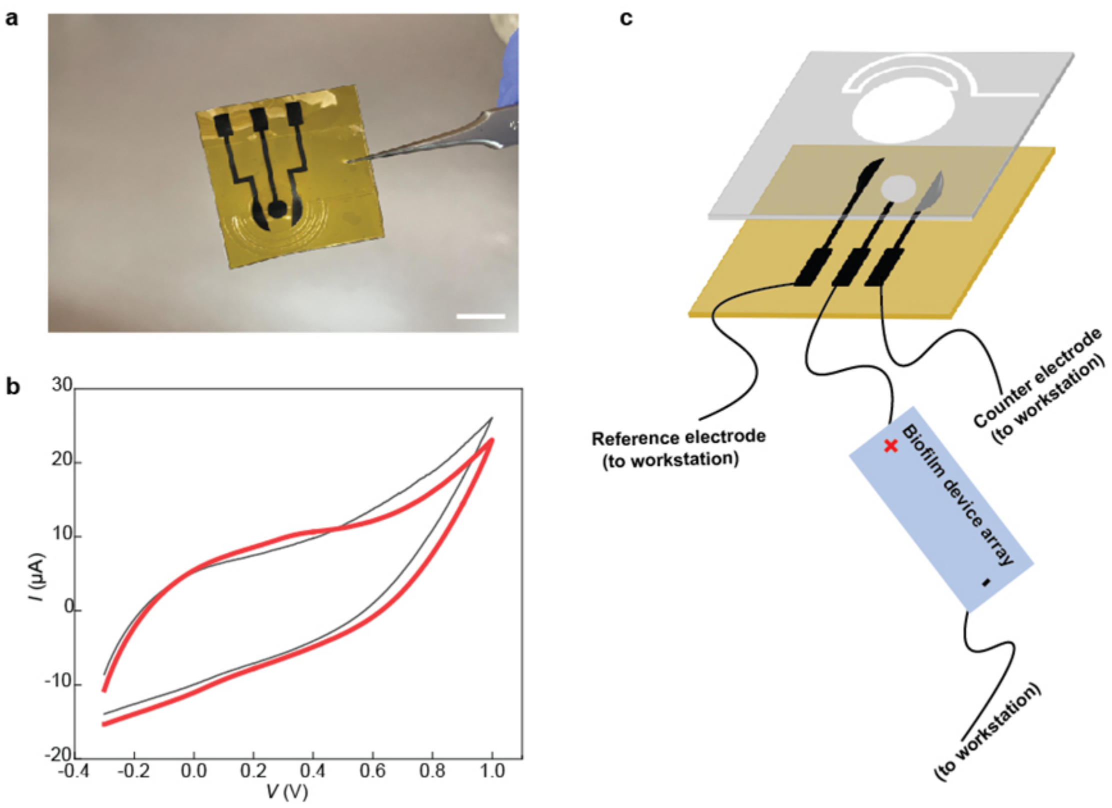

Supplementary Figure S19. **Biofilm-powered glucose sensor**. **a**, The glucose sensor consisted of laser-induced carbon electrodes on a polyimide substrate,[12,13] covered with a medical tape (MH-90445Q, Adhesives Research, Inc) with an engraved opening. Scale bar, 5 mm. Details of fabrication and functionalization processes can be found in *Methods*. **b**, Cyclic voltammetry (CV) measurement was first performed to identify the oxidization threshold voltage. CV from 500 μM glucose solution (red) showed oxidization peak ~0.4 V compared to reference (gray) from phosphate buffered saline solution without glucose. **c**, For real-time sensing, biofilm devices were connected to the working electrode to provide an effective voltage larger than 0.4 V. The three terminals were connected to an electrochemical workstation (CHI 440; CH Instruments) to acquire the current signal.

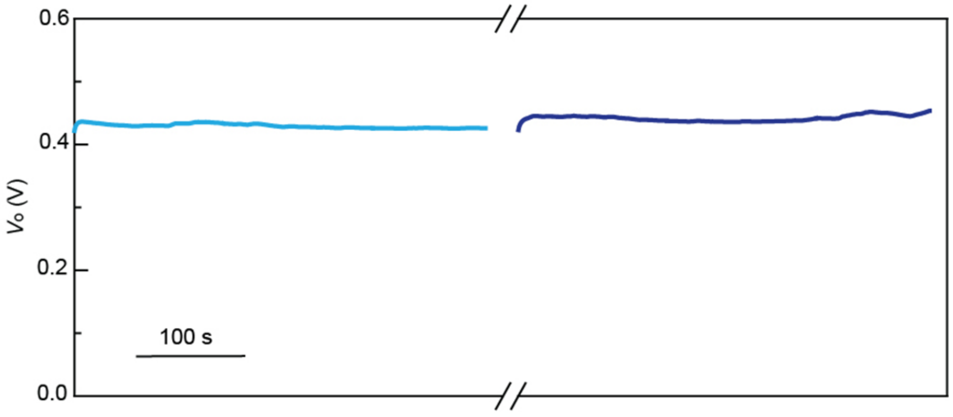

Supplementary Figure S20. Open-circuit voltage (V_o) measured from a biofilm device before (light blue) and after (dark blue) heating to 90°C for 30 min. The device had the same structure as in Supplementary Fig. S4.

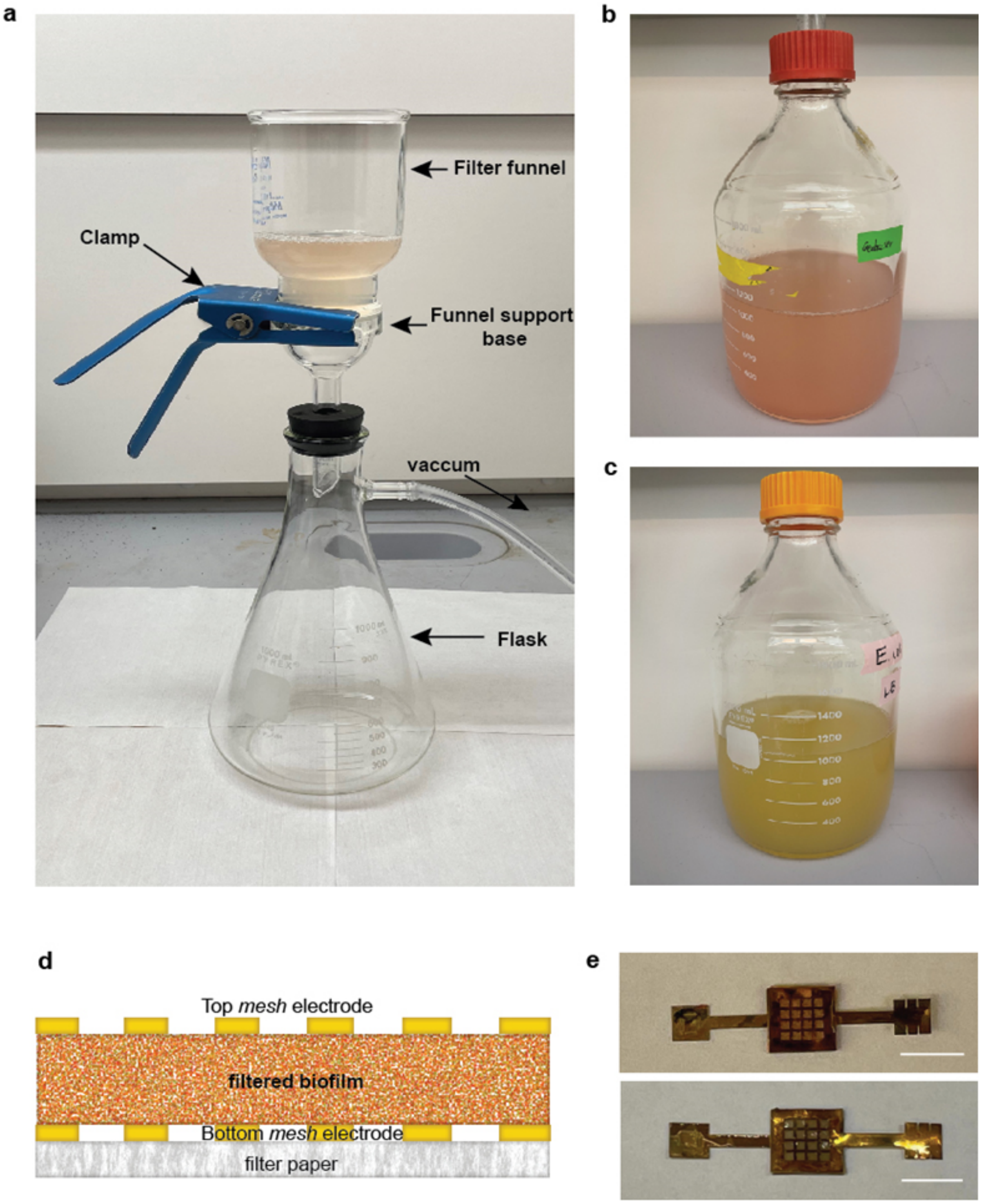

Supplementary Figure S21. **Mats of filtered cells and devices**. **a**, The setup of the filtering system. The microbial solution was passed through a papers filter (42.5 mm dia., 8 µm pore; Whatman) with vacuum filtration. A 300 ml volume was filtered repeatedly until the solution appeared to be clear. **b**, Prepared *G. sulfurreducens* solution for filtration. **c**, Prepared *Escherichia coli* solution for filtration. Details of the growth and solution preparation can be found in *Methods*. **d**, Cross-sectional schematic of the device made from filtered cells. The bottom mesh electrode was first placed on the filter paper before filtration. After filtration the top mesh electrode was capped. The filter paper was cut into the same size of the mesh electrode and left in the device, which would be in direct contact with water and hence did not block water evaporation. **e**, Fabricated devices using filtered biofilms of (top) *G. sulfurreducens* and (bottom) *E. coli*. Scale bars, 5 mm.

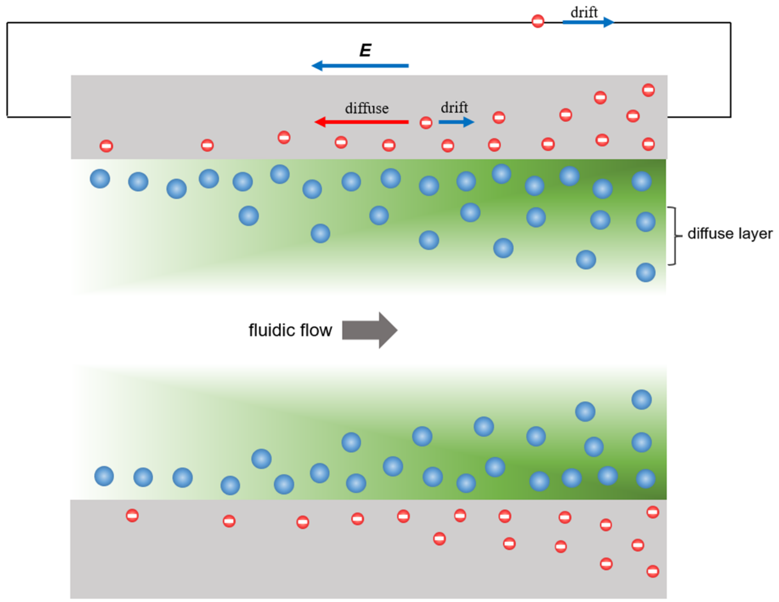

Supplementary Figure S22. **Image-electron model for current generation**.

It is generally acknowledged that the detailed streaming mechanism for current generation is not well known.[14] For example, the flow of ions in the channel leads to the expectation of ionic current, which then raises the question how the ionic current converts into external electron current (*i.e.*, what redox process may involve). The sustaining and enhanced current production from the biofilm-sheet device (Fig. 1d in main paper) provided opportunity for additional quest. Since the current production is largely independent of material composition in the biofilms (Fig. 4b in main paper) and ionic species/strengths (Fig. 3a in main paper), water-based redox which can yield gas release (*e.g.*, H_2, O_2) would be a more reasonable expectation. The 30-day continuous current (Fig. 1d in main paper) involved a total charge transfer of ~5.3 C, which would yield ~0.6 cm^3 gas volume (~1 $mm^3 \cdot h^{-1}$) that is observable. However, we did not observe bubbling on the immersed electrode for continuous (*e.g.*, 3 h) monitoring across different days. The results indicate that the current production may not involve redox process.

Based on above experimental evidence, we proposed an image-charge model (shown in above schematic) to account for the current generation. Positive ions (blue) in the diffuse layer, which is partially mobile and not charge neutral, are dragged by the flow to yield a charge gradient along the channel.[15] These ionic charges correspondingly induce a gradient in the image electrons (red) in the channel material. As a result, closed-loop current flow can be driven by the induced image-electron gradient without the need of redox process in the ions.

Specifically, the gradient in the electrons induces a diffusion current inside the channel material. An electric field (*E*) is built up to produce a drift current that counteracts the diffusion current at steady state (*e.g.*, open circuit). Connecting the two terminals effectively provides an additional route for the drift current. The portion split to the external route becomes the measured current. The mathematical description of above process can refer to Ref. 16 (Supplementary Fig. 16 therein) by taking electron as the diffusion species. The current flow can be sustained as long as the ionic charge gradient in the diffuse layer is sustained by the flow.

Supplementary References

1. Leang, C., Malvankar, N. S., Franks, A. E., Nevin, K. P. & Lovely, D. R. Engineering *Geobacter sulfurreducens* to produce a highly cohesive conductive matrix with enhanced capacity for current production. *Energy Environ. Sci.* **6**, 1901–1908 (2013).
2. Reppert, P. M & Morgan, F. D. Temperature-dependent streaming potentials: 2. Laboratory. *J. Geophys. Res.* **108**, 2547 (2003).
3. Pfitzner, J. Poiseuille and his law. *Anaesthesia* **31**, 273-275 (1976).
4. Wyman, Jr J. Measurements of the dielectric constants of conducting media. *Phys. Rev*. **35**, 623 (1930).
5. Fondriest Environmental, Inc. "Conductivity, Salinity and Total Dissolved Solids." https://www.fondriest.com/environmental-measurements/parameters/water-quality/conductivity-salinity-tds/
6. Kestin, J., Sokolov, M., & Wakeham, W. A. Viscosity of liquid water in the range -8 C° to 150 C°. *J. Phys. Chem. Ref. Data* **7**, 941-948 (1978).
7. van der Heyden, F. H. J., Bonthuis, D. J., Stein, D., Meyer, C. & Dekker, C. Electrokinetic energy conversion efficiency in nanofluidic channels. *Nano Lett*. **6**, 2232-2237 (2006).
8. http://hyperphysics.phy-astr.gsu.edu/hbase/Kinetic/relhum.html
9. https://www.weather.gov/epz/wxcalc_vaporpressure
10. Lu, Z. *et al*. Meniscus-guided coating of organic crystalline thin films for high-performance organic field-effect transistors. *J. Mater. Chem. C* **8**, 9133-9146 (2020).
11. Lee, T., Choi, Y. W., Lee, G., Kim, S. M., Kang, D. & Choi, M. Crack-based strain sensor with diverse metal films by inserting an inter-layer. *RSC Adv.* **7**, 34810-34815 (2017).
12. Lin, J. *et al*. Laser-induced porous graphene films from commercial polymers. *Nat. Commun*. **5**, 5714 (2014).
13. Yang, Y. *et al.* A laser-engraved wearable sensor for sensitive detection of uric acid and tyrosine in sweat. *Nat. Biotechnol*. **38**, 217-224 (2020).
14. Shen, D. *et al*. Moisture-enabled electricity generation: from physics and materials to self-powered applications. *Adv. Mater.* **32**, 2003722 (2020).
15. Kim, D. R., Lee, C. H. & Zheng, X. Probing flow velocity with silicon nanowire sensors. *Nano Lett*. **9**, 1984-1988 (2009).
16. Liu, X. *et al*. Power generation from ambient humidity using protein nanowires. *Nature* **578**, 550-554 (2020).
